# The relationship between health literacy and internet addiction among middle school students in Chongqing, China: A cross-sectional survey study

**DOI:** 10.1371/journal.pone.0283634

**Published:** 2023-03-24

**Authors:** Yang Liu, Nannan Wu, Jie Yan, Junjie Yu, Liping Liao, Hong Wang

**Affiliations:** School of Public Health, Research Center for Medicine and Social Development, Collaborative Innovation Center of Social Risks Governance in Health, Chongqing Medical University, Chongqing, China; Xiamen University - Malaysia Campus: Xiamen University - Malaysia, MALAYSIA

## Abstract

**Background:**

Internet addiction has emerged as a major global concern as a potential adverse impact of internet exposure on adolescents. Internet addiction is associated with many demographic variables; however, there is a lack of consensus on its relationship with health literacy. Therefore, the aim of the present study was to assess the rates of internet addiction and health literacy level among middle school students (grades 7 to 12) in Chongqing, China, as well as to investigate the association between them.

**Methods:**

A cross-sectional, questionnaire-based study was conducted among 8971 students who were randomly selected by using stratified cluster sampling between November and December 2019. The Internet Addiction Diagnostic Questionnaire, Adolescent Health Literacy Scale and a self-designed basic information questionnaire were used to collect data. Chi-square tests were performed to compare the differences in the distribution of internet addiction across health literacy levels as well as some sociodemographic characteristics. Multivariate logistic regression analyses were conducted to identify the association between health literacy and internet addiction.

**Results:**

The prevalence of internet addiction among middle school students in Chongqing was 6.1%. The percentage of the students who spent more than 4 hours online every day in the past week was 14.3%. In addition, 26.7%, 26.0%, 28.3% and 26.3% of the participants reported low functional, interactive, critical and total health literacy, respectively. After adjusting for the confounding effects of demographics, multivariate regression analysis showed that critical health literacy was a protective variable for internet addiction, while functional, and interactive health literacy were the risk variable (*P*<0.05). Furthermore, the internet addiction rates were higher among boys, students with good peer relationships, and students without parental supervision, whereas internet addiction rates were lowest among students in grade 12.

**Conclusions:**

The prevalence of internet addiction among middle school students in Chongqing is relatively high. Internet addiction is strongly negatively associated with critical health literacy, but it is positively associated with functional and interactive health literacy but not total health literacy. This study provides preliminary evidence for the predictive role of health literacy in internet addiction of adolescents.

## Introduction

With the exponential growth of information technology in the last two decades, the internet has become an integral part of our daily lives. Although the internet is useful for a variety of purposes, such as convenient electronic commerce, rapid sharing of information, contact with other cultures, emotional support and entertainment [1], the potential adverse effects of internet exposure on human health have emerged as a major global concern [2]. Since Dr. Kimberly Young published a case report in 1996, internet addiction, sometimes called internet dependence or problematic internet use (PIU), has been increasingly conceptualized as “a kind of psychopathological disorder” [3]. To date, there has been no agreement about the diagnostic criteria for internet addiction (IA). Specifically, such behaviors may include online gaming, gambling, shopping, pornography viewing, email checking, instant messaging and social media use [4]. IA, also called internet overuse, is common among adolescent and young adult populations, potentially leading to the weakness of performance in social activities and the ignoring of relationships in real life [5]. This behavioral dependence on the internet is a social problem. A previous study has reported that the estimated global prevalence of internet addiction in adults was 6.0% in 2014, ranging from 0.8% in Italy to 26.7% in Hong Kong [6]. Specifically, adolescents are a high-risk group for IA due to the enormous physical and psychological changes during puberty. According to a previous study of brain development, when maturational imbalances in the motivational system are the greatest during adolescence, adolescents may not be able to regulate motivational or emotional states in the same way as adults, which may explain the onset and elevated rates of addictive disorders during this developmental period [7]. Psychologically speaking, young people are interested in new technology and get used to the operation of such devices more easily than adults do. Adolescents, as digital natives, express their thoughts in an online space, try to keep up with fashion, use many kinds of applications (apps) and search for emotional relationships and support [1]. Some studies have examined the epidemiology of IA among adolescents, but findings vary greatly across countries due to different sampling methods, definitions, assessment instruments and sociocultural contexts [6]. For instance, the prevalence of IA ranges from 1.3% in Turkey [8] to 12.1% in Italy [3] using Young’s Internet Addiction Test (IAT), and the prevalence of IA ranges from 15.2% in Greece [9] to 17–26.8% in Hong Kong [10] using the Internet Addiction Diagnostic Questionnaire (IADQ). Moreover, the prevalence of IA is and 17.4% in Taiwan [11] using Chen’s Internet Addiction Scale (CIAS). Experts in the internet domain have all emphasized the addictive nature of the internet for adolescents, including college/university students [12–15].

A number of disorders have been identified as comorbidities of IA, including depression, attention-deficit hyperactivity disorder (ADHD), substance use, social anxiety disorder, aggressive behaviors and suicidal behavior [6, 16]. Moreover, certain demographic variables are also associated with IA, such as obesity, higher school grades, poor academic performance, engagement in high-risk behaviors, higher family income and lower level of parental attachment [17]. In April 2018, the Ministry of Education of China released an urgent notice concerning the prevention of primary and middle school students from indulging in the internet [18]. The opportunities and demand for teenagers to use electronic products have increased due to the COVID-19 pandemic, including the frequency and duration of recreational internet use and the rate of stay-up use (internet use after 00:00), leading to an increase in the rate of IA [19]. Exploring the variables associated with IA so that effective interventions can be taken is particularly important in promoting healthy adolescent growth under today’s epidemic prevention and control.

Health literacy (HL) has been defined and conceptualized in multiple ways. In the present study, we follow the World Health Organization’s definition of HL as follows: “Health literacy implies the achievement of a level of knowledge, personal skills and confidence to take action to improve personal and community health by changing personal lifestyles and living conditions” [20]. These are observable sets of skills that will vary from individual to individual. According to Nutbeam’s health literacy theory, HL is divided into functional, interactive and critical health literacy. Such a classification has the advantage of signaling the impact that differences in skill levels may have on health-related decisions and actions and it helps to distinguish between interventions that are task-based or skills-based [21]. Functional health literacy is the most common type of health literacy, which describes basic level skills that are sufficient for individuals to obtain relevant health information (for example, on health risks and how to use the health system) and to be able to apply that knowledge to a range of prescribed activities [21]. Interactive health literacy describes more advanced literacy skills that enable individuals to extract health information and derive meaning from different forms of communication, to apply new information to changing circumstances and to engage in interactions with others to extend the information available and make decisions [21]. Critical health literacy describes the most advanced cognitive skills that can be applied to critically analyze information from a wide range of sources and information relating to a greater range of health determinants as well as to use this information to exert greater control over life events and situations that impact health [21].

Currently, many tools to measure health literacy have been validated for use in adolescent populations. However, concern has been expressed that most tools are not comprehensive, measuring only selective domains that are thought to be markers of an individual’s overall capacity and focusing on functional health literacy [22], and their applicability is limited. For example, the Rapid Estimate of Adolescent Literacy in Medicine (REALM-Teen) and Newest Vital Sign (NVS) are administered by an interviewer to assess functional health literacy [23]. The eHealth Literacy Scale (eHEALS) is a self-completed measure of perceived electronic health literacy skills [24], and Media Health Literacy includes the categories of identification, critical evaluation, influence and action [25]. In China, although the Chinese Citizen Health Literacy Questionnaire (China Health Education Center, 2012) is widely used in adult populations [26], health literacy among middle school students has been less studied as this questionnaire may not be applicable to adolescents due to the physiological and psychological differences between adolescents and adults. It is highly likely that different measurement tools will be required for different ages and stages in life even if the structure of the concept remains constant.

According to addictive behavior theory, addictive behaviors are mainly divided into substance-related addiction (i.e., smoking, alcohol consumption and drug use) and nonsubstance addiction (i.e., internet usage, video gaming, gambling, sex and shopping). It is conjectured that similar mechanisms and changes are involved in different addictions [7]. The addictive process involves problems with aberrant reward systems and impulsivity. In terms of the reward system theory, previous research has shown that neural circuits in the brain involving reward are hijacked and rewired during the process of addiction; specifically, dopaminergic projections to the nucleus accumbens have been implicated [16]. Dopamine increases in the nucleus accumbens with the administration of drugs of abuse or certain behaviors (gaming, gambling and sexual behavior) [7]. Similar to substance-related addiction, the development of internet or gaming addiction is characterized by an overall reward deficiency that entails decreased dopaminergic activity at the molecular level [27]. In addition, risk behaviors do not occur in isolation; instead, they tend to be positively interrelated among themselves and coexist as lifestyle practices due to their similar psychological meanings and functions [28]. One large-scale study among teenagers has clarified the correlation between internet addiction and substance abuse, specifically smoking and drug use [29]. Previous findings have revealed that adolescent health literacy is significantly associated with some substance-related addictive behaviors, such as smoking, drinking and drug use [23, 30]. Moreover, negative correlations were found between HL and screen time [31]. Because screen time in excess of 4 hours per day is a requirement for the diagnosis of internet addiction, we speculate that health literacy is related to internet addiction.

In terms of reward impulsivity theory, impulsivity leads to an emphasis on short-term consequences without consideration for longer-term consequences. In IA, there is a tendency to discount rewards rapidly and perform poorly on decision-making tests. Individuals with IA are more likely to have high trait impulsivity [16]. A recent review has revealed that lower HL levels in adolescents is related to worse ability to make rational decisions (decisions based on perceived risk-reward tradeoffs), thus making adolescents more likely to make reactive decisions (decisions based on immediate and direct judgments about the environment) [31]. Adolescents’ goals are more likely to maximize immediate pleasure, such as drinking and drug use [32]. Therefore, both low health literacy and internet addiction are linked to poor decision-making skills.

Based on the addictive behavior theory described above, it is reasonable to hypothesize that there may be an association between internet addiction and health literacy. Compared to smoking and alcohol consumption, the onset of internet addiction or pathological internet use is more insidious and can pose long-term health and social hazards. However, little is known about the relationship between HL and IA among adolescents. Among several variables that may be related to IA among adolescents, HL has been identified as a modifiable factor that can be improved. Therefore, a better understanding of this relationship would be useful in developing future IA interventions [33]. Therefore, the present study aimed to examine the prevalence of IA and to identify whether there is an association between HL and IA among teenagers. We used the Adolescent Health Literacy Scale designed by our team to fill this gap to a certain extent. The study was conducted with junior high and high school students in four representative districts of Chongqing (n = 8971) to indirectly monitor the HL and IA of middle school students in Chongqing.

In this context, the following research objectives were proposed: 1) validate the Adolescent Health Literacy Scale; and 2) to investigate the link between internet addiction and health literacy.

## Methods

### Study design and participants

**Fig 1:**
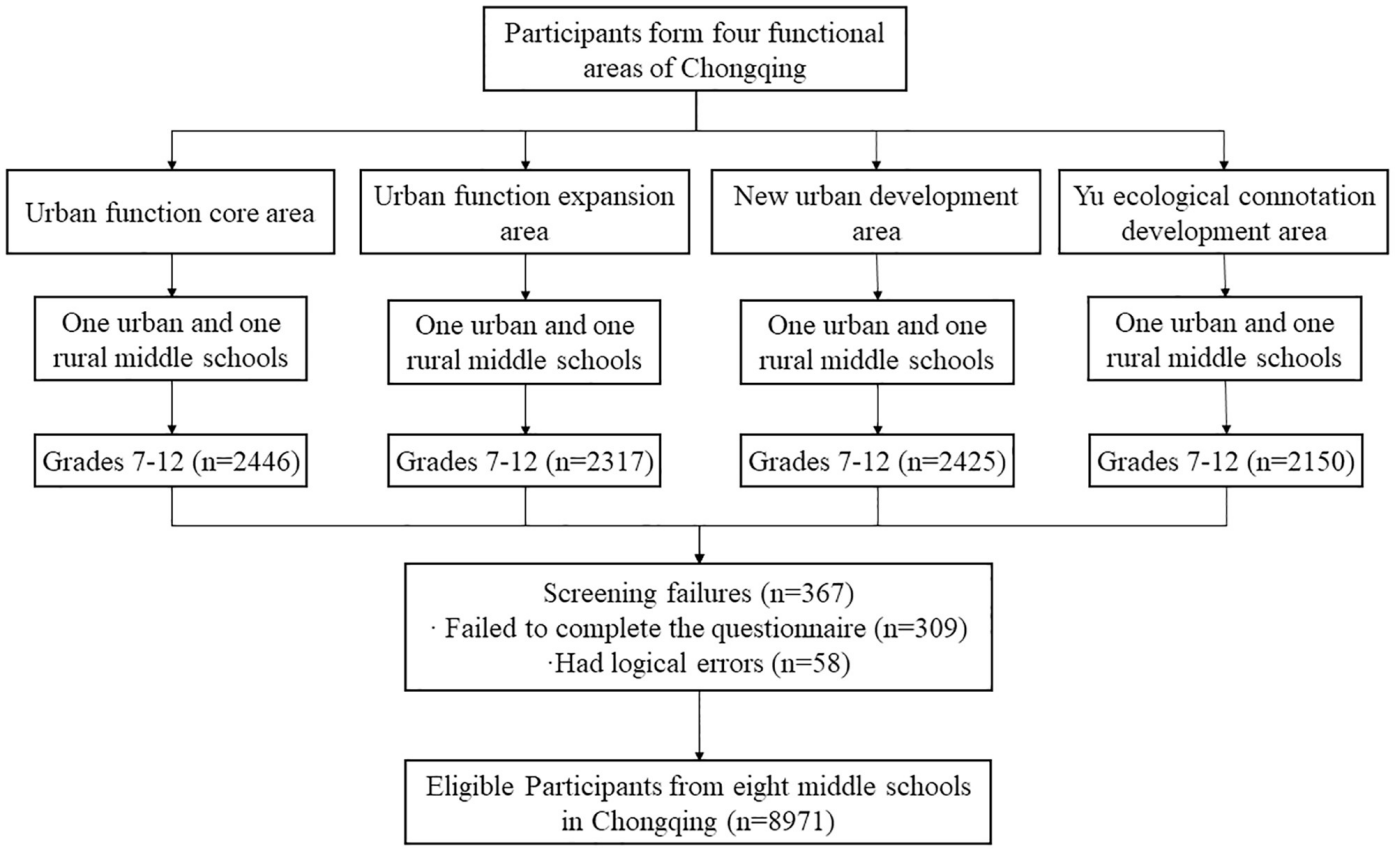
Stratified cluster sampling was adopted to identify participants in this study.

Data were collected between November and December 2019. Stratified cluster sampling was used to obtain a representative sample of participants. In the first stage, one district was randomly selected from each of the four different functional areas of Chongqing, China. In the second stage, one urban full-time secondary school and one rural full-time secondary school (including junior and senior high schools) were randomly selected from each district. Four classes were then randomly chosen for each grade from grades 7 to 12 in the eight selected schools. The participants were students in these classes who had the ability to complete the questionnaire independently. Questionnaires with incorrect or missing data (absence rate ≥10%) were excluded. Finally, a total of 9338 questionnaires were collected and 8971 were valid, yielding an effective response rate of 96.7% (Fig 1). The present study was approved by the Ethical Committee of Chongqing Medical University in accordance with the Declaration of Helsinki, and written informed consent was obtained from students and their parents before the research study began. No personal identifying information was collected from the participants.

### Measures

#### Sample size

The required sample size was calculated using the following formula:

$$N=\frac{Z_{\alpha /2}^{2}p\left(1-p\right)}{\delta^{2}}$$

A previous study has reported that the rate of internet addiction among middle school students in Chongqing is 9.49% [34]. Therefore, the required sample size was calculated based on p = 0.095, α = 0.05, Z_α/2_ = 1.96 and δ = 0.01, resulting in a required sample size of 3303 in the present study. To control for invalid survey samples, we increased the sample size by 20%, resulting in 3964 as the minimum sample size required for the present study.

#### Sociodemographic characteristics

A self-administered Basic Information Questionnaire was designed to collect the following information: residence (rural/urban), gender (male/female), grade (grades 7 to 12), only child in family or not (yes/no), family income (five options), school achievement (five options), academic pressure (five options) and peer relationship (five options). The five options for the above four variables were poor, comparatively poor, moderate, comparatively good and good. The four items (family income, school performance, academic pressure and peer relationships) required participants to select the most appropriate one based on their subjective feelings without repeated reflection, using the status of their classmates as a reference [35].

#### Internet addiction

The Internet Addiction Diagnostic Questionnaire (IADQ), taken from the Chinese Adolescent Health Risky Behavior Questionnaire (Junior Middle School Part), is a standard questionnaire (S1 File). The IADQ includes 10 items; when the student meets the criteria for the first item (in the past 7 days, surfing the internet more than 4 h per day) and meets at least four of the other nine items, he or she is identified as an internet addict [36]. The Cronbach’s α of the scale was 0.87 in the present study. The validity and reliability of this instrument have been confirmed by our previous study [34].

#### Adolescent health literacy

The Adolescent Health Literacy Scale (AHLS) was developed by our group based on the original Health Literacy Scale for Middle School Students [37, 38]. In the present study, we improved this scale by adding some items to make it more applicable to junior high and high school students (S2 File). The newly improved AHLS includes 12 factors in 3 dimensions, namely, functional health literacy (37 items), interactive health literacy (14 items) and critical health literacy (10 items). The 61 items are rated on a five-point Likert scale (strongly disagree, disagree, unsure, agree and strongly agree; or completely inconsistent, inconsistent, unclear, consistent or completely consistent), and the test scores range from a minimum of 61 points to a maximum of 305 points; a high score indicates that the student’s health literacy level is high. The HL levels of the respondents were classified into the following three levels using quartiles as the cutoff points: high (total HL>272, functional HL>169, interactive HL>62 and critical HL>48), moderate (total HL = 272–235, functional HL = 144–169, interactive HL = 49–62 and critical HL = 39–48) and low (total HL<235, functional HL<144, interactive HL<49 and critical HL<39) [38, 39]. We tested the reliability, validity and feasibility of this scale in the present research sample. The test-retest reliability of the total, functional, interactive and critical health literacy was 0.619–0.759. The internal consistency (Cronbach’s α) was 0.886–0.939, and the split-half reliability was 0.497. The correlation coefficients between the different dimensions were 0.395–0.560, and the correlations were 0.698–0.864 between the dimensions and the total scale. The exploratory factor analysis (KMO = 0.922, *P*<0.001) confirmed that the scale consisted of three core dimensions, namely, functional, interactive and critical health literacy. In the present study, the Cronbach’s α of total, functional, interactive and critical health literacy was good (0.880<α<0.915).

### Statistical analysis

All data were entered into the Epidata 3.1 database and analyzed with SPSS 23.0 (SPSS Inc. Chicago, USA). First, frequencies and proportions for categorical variables were used to describe the characteristics of the participants and to describe IA among the participants by different characteristics. Second, chi-square tests were used to examine whether internet addicts and noninternet addicts differed in HL level and sociodemographic characteristics (i.e., residence, gender, grade, only child in family, family income, school achievement, academic pressure and peer relationship). Intergroup comparisons were made using chi-square splitting (*P* values were Bonferroni corrected, α’ = 2α/[k*(k-1)]). Third, the forward stepwise-Wald method was used in multivariate logistic regression analysis. Multiple logistic regression was conducted to determine the associations between HL level and IA. Internet addiction was considered the outcome variable, and health literacy level was considered the independent variable. The correlation intensity was expressed by *OR* and 95% *CI*. After determining the absence of collinearity, three models were built. In the crude model, we examined the association between IA and total HL, as well as three dimensions of HL. In Model 1, we additionally adjusted for several variables that were statistically significant in the univariate analysis (i.e., grade, peer relationships, and parental supervision). In Model 2, we additionally adjusted for other variables included in the univariate analysis (i.e., residence, gender, only child in family, family income, school achievement and academic pressure). The value of ɑ was equal to 0.05.

## Results

### Participant characteristics

Among the 8739 participants, the gender ratio of the participants was close to 1:1 (female: 52.2%). Regarding location, 52.6% of the students were from urban areas, and 47.4% of the students were from rural areas. The participants’ ages ranged from 10.10 to 20.96 years (15.30 ± 1.78). The proportions of students studying in grades 7, 8, 9, 10, 11 and 12 were 16.3%, 16.2%, 15.8%, 17.4%, 17.1% and 17.1%, respectively. Most of the students (67.6%) were not only child and 70.8% of the students came from a household with moderate income. Almost half of the students (42.8%) perceived their academic pressure as moderate, and 37.1% of the students considered their school achievement to be comparatively good. More than half of the participants (50.6%) had comparatively good relationships with their classmates and friends. Most parents (72.4%) limited the time their children spend watching TV, playing with cell phones or playing video games each day (Table 1).

**Table 1:** Distribution of sociodemographic characteristics between the internet addiction group and the noninternet addiction group (n = 8739).

	IA ^a^ (n = 529)	NIA ^b^ (n = 8210)	Total (n = 8739)	*χ* ^*2*^	*P*
**Residence**				0.544	0.472
Urban	270 (5.9)	4326 (94.1)	4596 (52.6)		
Rural	259 (6.3)	3884 (93.7)	4143 (47.4)		
**Gender**				2.64	0.104
Male	271 (6.5)	3907 (93.5)	4178 (47.8)		
Female	258 (5.7)	4303 (94.3)	4561 (52.2)		
**Grade**				46.26	<0.001
Seven	69 (4.8)	1354 (95.2)	1423 (16.3)		
Eight	84 (5.9)	1336 (94.1)	1420 (16.3)		
Nine	103 (7.4)	1281 (92.6)	1384 (15.8)		
Ten	124 (8.1)	1400 (91.9)	1524 (17.4)		
Eleven	104 (6.9)	1393 (93.1)	1497 (17.1)		
Twelve	45 (3.0)	1446 (97.0)	1491 (17.1)		
**The only child**				0.06	0.811
Yes	174 (6.1)	2659 (93.9)	2833 (32.4)		
No	355 (6.0)	5551 (94.0)	5906 (67.6)		
**Family income**				3.44	0.488
Poor	8 (3.7)	206 (96.3)	214 (2.4)		
Comparatively poor	65 (6.3)	973 (93.7)	1038 (11.9)		
Moderate	383 (6.2)	5804 (93.8)	6187 (70.8)		
Comparatively good	64 (5.9)	1029 (94.1)	1093 (12.5)		
Good	9 (4.3)	198 (95.7)	207 (2.4)		
**School achievement**				3.87	0.425
Poor	71 (7.2)	918 (92.8)	989 (11.3)		
Comparative poor	126 (5.6)	2140 (94.4)	2266 (25.9)		
Moderate	203 (6.3)	3043 (93.7)	3246 (37.1)		
Comparative good	110 (5.7)	1823 (94.3)	1933 (22.1)		
Good	19 (6.2)	286 (93.8)	305 (3.5)		
**Academic pressure**				8.11	0.088
High	71 (7.8)	844 (92.2)	915 (10.5)		
Comparatively high	213 (5.8)	3482 (94.2)	3695 (42.3)		
Moderate	226 (6.0)	3516 (94.0)	3742 (42.8)		
Comparatively low	9 (3.6)	239 (96.4)	248 (2.8)		
Low	10 (7.2)	129 (92.8)	139 (1.6)		
**Peer Relationship**				47.16	<0.001
Poor	2 (4.4)	43 (95.6)	45 (0.5)		
Comparatively poor	10 (6.0)	157 (94.0)	167 (1.9)		
Moderate	85 (3.8)	2176 (96.2)	2261 (25.9)		
Comparatively good	269 (6.1)	4153 (93.9)	4422 (50.6)		
Good	163 (8.8)	1681 (91.2)	1844 (21.1)		
**Parental control**				0.066	0.011
Yes	343 (5.4)	5980 (94.6)	6323 (72.4)		
No	186 (7.7)	2230 (92.3)	2416 (27.6)		

^a^ IA: internet addiction group.

^b^ NIA: noninternet addiction group.

Quantitative data are expressed by “n (%)”.

### Prevalence of internet addiction

More than half (50.6%) of the students admitted that they spent more than one hour online every day (i.e., cell phones, iPads and other electronic devices) in the past week, and 14.3% of the students spent more than 4 hours. The prevalence of IA among middle school students in Chongqing was 6.1%. Moreover, a significant difference was observed in the prevalence of internet addiction for grade, peer relationship and parental control (*P*<0.05). In addition, the rate of IA was higher in grade 10 than in grade 7, and the rate of IA was lowest in grade 12 compared to grades 8–11 (*P*<0.003) with no significant difference in the remaining grades. The rates of IA in the three groups with moderate, comparatively good and good peer relationships were significantly different when compared to each other with the lowest in the moderate group and the highest in the good group (*P*<0.05); however, no significant differences were found in the remaining groups. In addition, there were no significant differences in gender, only child status, family income, school achievement or academic pressure between the internet addiction and noninternet addiction groups (Table 1).

### Current levels of health literacy

**Fig 2:**
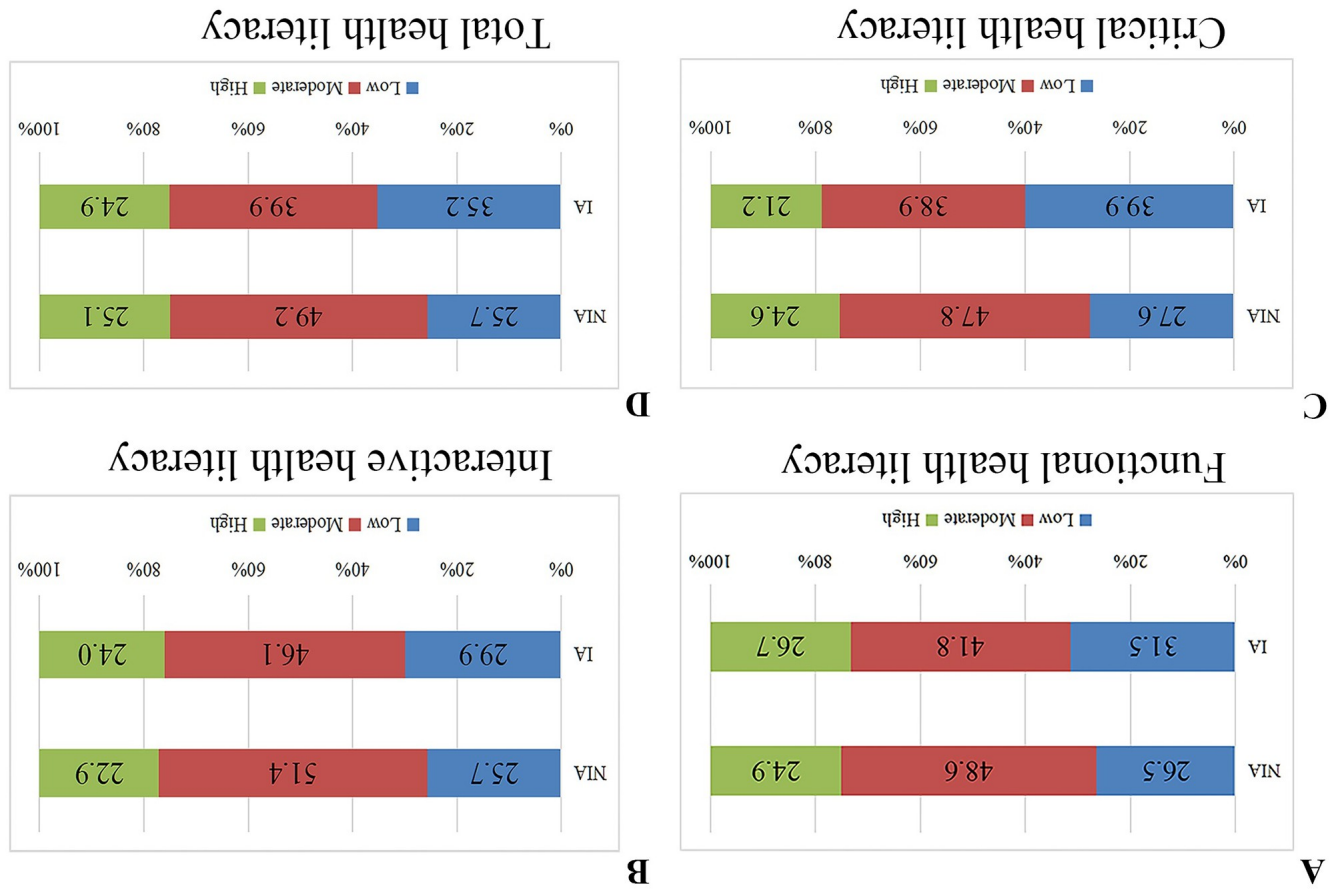
Proportions of high, moderate, and low health literacy between IA and NIA groups across different dimensions (%).

Among the 8739 participants, 48.6%, 48.2%, 51.0% and 47.3% of the students had moderate levels of total HL, functional HL, interactive HL and critical HL, respectively. Moreover, 26.3%, 26.8%, 26.0% and 28.3% of the students had low levels of total HL, functional HL, interactive HL and critical HL, whereas 25.1%, 25.0%, 23.0% and 24.4% of the students had high levels of total HL, functional HL, interactive HL and critical HL, respectively. The differences in different levels of health literacy between the internet addiction and noninternet addiction groups are shown in Fig 2. Significant differences were found between the internet addiction and noninternet addiction groups in all three dimensions and total health literacy levels (all *P*<0.05). Furthermore, intergroup comparisons showed that the IA rates were higher in the group with lower levels of functional and interactive HL than in the group with middle levels (Table 2), and the IA rates were higher in the group with lower levels of critical and total HL than in the group with middle and high levels (all *P*<0.017).

**Table 2:** Comparison of health literacy across different dimensions between IA and NIA groups.

	IA (n = 529)	NIA (n = 8210)	Total	*χ* ^*2*^	*P*
**Functional health literacy**				10.25	0.006
Low ^a^	167 (7.1)	2174 (92.9)	2341 (26.8)		
Moderate	221 (5.2)	3992 (94.8)	4213 (48.2)		
High	141 (6.5)	2044 (93.5)	2185 (25.0)		
**Interactive health literacy**					
Low ^a^	158 (7.0)	2112 (93.03)	2270 (26.0)	6.20	0.045
Moderate	244 (5.5)	4216 (94.5)	4460 (51.0)		
High	127 (6.3)	1882 (93.7)	2009 (23.0)		
**Critical health literacy**				37.28	<0.001
Low ^b^	211 (8.5)	2264 (91.5)	2475 (28.3)		
Moderate	206 (5.0)	3927 (95.0)	4133 (47.3)		
High	112 (5.3)	2019 (94.7)	2131 (24.4)		
**Total health literacy**				25.69	<0.001
Low ^b^	186 (8.1)	2112 (91.9)	2298 (26.3)		
Moderate	211 (5.0)	4039 (95.0)	4250 (48.6)		
High	132 (6.0)	2059 (94.0)	2191 (25.1)		

^a^ The difference was statistically significant when compared to the moderate level.

^b^ The difference was statistically significant when compared to the moderate or high level. α = 0.05.

Quantitative data are expressed by “n (%)”

### Association between internet addiction and health literacy

Unlike the results of the univariate analysis, the results of the multivariate regression showed that internet addiction was positively associated with functional and interactive HL but negatively associated with critical HL (*P*<0.05, Table 3). Compared to high HL, moderate functional HL, low and moderate interactive HL were protective factors for IA, but low critical HL was a risk factor (*P*<0.05). After adjusting for grade, peer relationships and parental supervision, the adjusted *ORs* (*aORs*) of IA were almost unchanged, and the association between IA and HL was the same as that in the crude model. Significant associations were also found between IA and both peer relationships and parental supervision. Compared to the high group, the rate of IA was lower in the moderate/comparative good group. Compared to grade 12, students in grades 7–11 all had higher rates of internet addiction. Furthermore, the *aORs* did not change substantially from Model 1 to Model 2, which additionally adjusted for residence, gender, only child in family, family income, school achievement, and academic pressure. Male sex was a risk factor for IA, and other covariates were not associated with IA. Generally, IA was associated with a higher risk of high functional and interactive HL but a low critical HL. IA rates were higher among males, students with good peer relationships and students without parental supervision, and IA rates were lowest among students in grade 12.

**Table 3:** Multivariate logistic regression of internet addiction.

Variable	Crude model				Model 1^a^				Model 3^b^			
	*β*	*SE*	*OR* (95% *CI*)	*P*	*β*	*SE*	*aOR* (95% *CI*)	*P*	*β*	*SE*	*aOR* (95% *CI*)	*P*
**Functional HL** ^c^												
Low	-0.410	0.214	0.66 (0.44–1.01)	0.055	-0.386	0.214	0.68 (0.45–1.03)	0.071	-0.384	0.214	0.68 (0.45–1.04)	0.073
Moderate	-0.385	0.170	0.68 (0.49–0.95)	0.023	-0.376	0.170	0.69 (0.49–0.96)	0.027	-0.376	0.170	0.69 (0.49–0.96)	0.027
**Interactive HL** ^c^												
Low	-0.429	0.181	0.65 (0.46–0.93)	0.018	-0.450	0.183	0.64 (0.45–0.91)	0.014	-0.461	0.184	0.63 (0.44–0.90)	0.012
Moderate	-0.322	0.143	0.72 (0.55–0.96)	0.024	-0.312	0.146	0.73 (0.55–0.97)	0.032	-0.319	0.146	0.73 (0.55–0.97)	0.029
**Critical HL** ^c^												
Low	0.712	0.176	2.04 (1.44–2.88)	<0.001	0.680	0.178	1.97 (1.39–2.80)	<0.001	0.686	0.179	1.99 (1.40–2.82)	<0.001
Moderate	0.204	0.149	1.23 (0.92–1.64)	0.171	0.183	0.151	1.20 (0.89–1.61)	0.224	0.195	0.151	1.22 (0.90–1.63)	0.198
**Total HL** ^c^												
Low	0.445	0.280	1.66 (0.90–2.70)	0.112	0.464	0.281	1.59 (0.92–2.76)	0.099	0.461	0.282	1.59 (0.91–2.75)	0.102
Moderate	0.054	0.204	1.06 (0.71–1.57)	0.793	0.058	0.205	1.06 (0.71–1.58)	0.775	0.044	0.206	1.05 (0.70–1.56)	0.832
**Grade** ^c^												
Seven	-	-	-	-	0.642	0.198	1.90 (1.29–2.80)	0.001	0.656	0.199	1.93 (1.31–2.85)	0.001
Eight	-	-	-	-	0.863	0.191	2.37 (1.63–3.44)	<0.001	0.877	0.191	2.40 (1.65–3.50)	<0.001
Nine	-	-	-	-	1.058	0.184	2.88 (2.01–4.14)	<0.001	1.070	0.185	2.92 (2.03–4.19)	<0.001
Ten	-	-	-	-	1.112	0.179	3.04 (2.14–4.32)	<0.001	1.113	0.180	3.04 (2.14–4.33)	<0.001
Eleven	-	-	-	-	0.901	0.183	2.46 (1.72–3.53)	<0.001	0.902	0.184	2.46 (1.72–3.53)	<0.001
**Peer relationship** ^c^												
Poor	-	-	-	-	-0.635	0.734	0.53 (0.13–2.23)	0.387	-0.776	0.744	0.46 (0.11–1.98	0.297
Comparatively poor	-	-	-	-	-0.389	0.340	0.68 (0.35–1.32)	0.253	-.497	0.344	0.61 (0.31–1.20)	0.149
Moderate	-	-	-	-	-0.877	0.140	0.42 (0.32–0.55)	<0.001	-0.938	0.144	0.39 (0.30–0.52)	<0.001
Comparatively good	-	-	-	-	-0.393	0.105	0.68 (0.55–0.83)	<0.001	-0.416	0.107	0.66 (0.54–0.81)	<0.001
**Parental supervision** ^d^												
No	-	-	-	-	0.382	0.095	1.47 (1.22–1.77)	<0.001	0.435	0.098	1.55 (1.27–1.87)	<0.001
**Gender** ^e^												
Male	-	-	-	-	-	-	-	-	0.207	0.094	1.23 (1.02–1.48)	0.028

^a^ Model 1 additionally adjusted for grade, peer relationships, and parental supervision.

^b^ Model 2 additionally adjusted for residence, gender, only child in family, family income, school achievement and academic pressure.

^c^ Highest level was the reference value.

^d^ Yes was the reference.

^e^ Female was the reference.

## Discussion

### Principal findings

The present study included 8739 middle school students in Chongqing, and the rate of internet addiction (IA) was 6.1%, which was lower than the rate of IA in Tianjin (6.92%) but higher than that in Shanghai (2.3%) and Suzhou (1.56%) using the same assessment tools and criteria [40–42]. These differences may be related to the different levels of socioeconomic development in different regions. A previous study has reported a significantly higher frequency and duration of electronics use and self-ratings of addiction to electronics among both IA and NIA individuals during the COVID-19 pandemic than before as well as a higher incidence of depression, anxiety and stress states above a mild level [19]. This previous study prompted local health and education departments to develop prevention and intervention programs based on the current status of local youth internet addiction and the development characteristics of the region.

It has been demonstrated that IA is more prevalent in adolescents than in other age groups [43]. It is apparent that the etiology of IA is rather complex, and there is a range of possible risk factors that may influence the onset and development of the problem. As there are many proposed definitions, different conceptual frameworks have also been proposed as the theoretical basis for the understanding of IA, particularly among adolescents [44, 45]. Among these theoretical models, stress or anxiety reduction has been proposed as a possible explanatory theory for IA [43]. According to this theory, the motivation for the behavioral maintenance of IA of “overusers” is that the internet is used as a means for stress or tension reduction [43]. Another theory that has also received much attention, especially for IA among adolescents, is the problem behavior theory [46, 47]. The problem behavior theory advocates that there are three main systems, namely, the personality, environment and behavioral systems, in the conceptual structure of any problematic behavior in young people [48]. The propensity of any involvement in problematic behaviors is determined by the balance among risk and protective factors in the three systems [48]. In recent years, many scholars have focused on the risk and protective factors of adolescent internet addiction, including adverse childhood experiences (ACEs), traumatic experiences and peer attachment [11, 12, 49–51]. In addition, the mediating role of internet addiction has become a hot topic of recent research. Mlouki et al. found that internet addiction mediates the relationship between ACEs and sleep disorders among adolescents [52].

In early 2008, China issued the "Chinese citizens’ health literacy—Basic Knowledge and Skills (Trial)", which is the first government document to define citizens’ health literacy in the world [53]. In the present study, we found that 26.3% of the students in Chongqing presented with a low level of total HL. After categorizing the health literacy into three dimensions, we also found that functional and interactive HL were positively correlated with IA but that critical HL was negatively correlated with IA when analyzing IA-related variables in the regression analysis. These findings implied that students with high functional and interactive HL but low critical HL were more likely to obtain IA than their counterparts after adjusting for relevant demographic variables. Because no gold standard exists for measuring health literacy, studies differ not only in the tools used but also in specifications of thresholds for distinguishing between health literacy levels. Thus, the results of the present study should be interpreted with caution.

In agreement with our hypothesis, critical HL was a protective variable of IA, implying that adolescents who are accustomed to making careful judgments and considerations about the reliability, accuracy and applicability of the health information they receive may be less likely to become addicted to the internet [54]. The results of the present study emphasized the importance of critical health literacy. However, most of the recent studies focus on total health literacy or functional health literacy, and no studies have reported on critical health literacy. Only a few studies have addressed similar concepts. The emphasis of Levin-Zamir et al. on critical analysis and action in media health literacy is most consistent with critical health literacy [25]. Based on a study of IA, Wegmann et al. suggested that improving personal skills, such as high information literacy, may be a preventive factor for using virtual networks and the internet [55]. These findings suggest that improving the critical HL of adolescents may be an effective strategy to prevent the incidence of adolescent IA to some extent, but future studies are required to confirm this hypothesis. Interestingly, students with high functional health literacy reported significantly higher rates of IA than those with moderate levels, while differing little from students with low levels. Thus, adolescents with moderate functional HL may be the least likely to become addicted to the internet. In contrast to our hypothesis, high interactive HL was also a risk variable for IA, and students at a high level reported higher rates of IA than those at a moderate or low level.

Furthermore, total health literacy was not associated with internet addiction. In contrast, Tang showed that students with low health literacy are more likely to be addicted to the internet than those with high health literacy [56]. Similarly, Rong indicated that military college students who play online games for more than 5 hours weekly have lower health literacy levels [26]. There are few studies on health literacy and internet addiction, and similar studies on health literacy and other health-related risk behaviors have mostly been on total health literacy and not explored by dimensions. The present findings showed that the strength of the association and even the direction of the association between different dimensions of health literacy and internet addiction were not identical, suggesting that exploring only total health literacy may obscure the differences between dimensions. Health literacy is worthy of further analysis regarding its value in improving adolescent health and ultimately adult health [23]. Unlike previous related studies, the present findings showed that critical health literacy was positively associated with internet addiction, while functional or interactive health literacy was the opposite. Yang found that individuals with low interactive HL are more likely to have too much screen time [31]. Similarly, a previous study has reported that functional HL is an independent predictor of a total health-promoting behavior score [57]. Currently, with the highly developed internet technology, information is easy to access, and access to health knowledge is not lacking as before. More often, most students are forced to accept information, and it is noteworthy that not all of this information is beneficial to adolescent development. Therefore, it is especially important to critically assess whether this information is beneficial, and to selectively accept beneficial information. Thus, critical HL may be more important than functional or interactive HL, and it is worthy of further analysis regarding its value in improving adolescent health and ultimately adult health. However, it is likely that each dimension will require different intervention strategies and have different effects on IA. Future studies should explore the dimensions simultaneously to determine whether they represent a single concept and their differential impact on IA in adolescents.

In addition to health literacy, a significant difference was observed in the prevalence of IA for gender, grade, peer relationship and parental supervision. The male sex was a risk factor for IA, which was consistent with previous studies [51, 58]. This may be related to the time spent online; girls focus on playing time-killing games, socializing, texting and shopping online, which have shorter durations than online gaming, whereas boys prefer massive multiplayer online role-playing games (MMORPGs) and violent games, resulting in longer online screen times [19]. A possible reason for this is that girls have better self-control and emotional regulation, and they mature physically and mentally earlier than boys, which can reduce pathological internet use, especially when negative events occur [59]. Peer relationships are one of the main relationships of adolescents and are important for their existence, social identity and social development. Several studies have found that peer relationships are a protective variable in adolescent IA; the more dissatisfied and anxious adolescents are about peer interactions, the more likely they are to develop internet addiction [49, 50]. In contrast, our previous study indicated that respondents with moderate relationships have the lowest rate of IA, while those with good relationships have the highest rate [36]. Thus, IA may be a group aggregation phenomenon, and internet addicts are more likely to communicate with classmates who share the same hobbies and likely to influence his or her friends to also become internet addicted through daily life [60]. Students with moderate peer relationships may be more socially rational [61]. However, more research is needed to verify this hypothesis. As we expected, parental supervision is a protective factor. Teenagers have poor self-control ability and will increase their time and frequency of internet access without restraint. Previous studies have indicated that parents limiting students’ time spent watching TV and playing games may help reduce the rate of IA [62, 63]. Of the six grades, grade 12 had the lowest rate of internet addiction with little difference in the other grades, which may have been due to the pressure to advance to higher education leading individuals in grade 12 to spend most of their time studying and less time using electronics, thus reducing the incidence of IA. Given that parental supervision was also associated with IA, we speculated that perhaps parental supervision or time spent online was a moderating variable between grade and IA, but due to the limitations of this scale, we did not collect the time spent online by the respondents, thus warranting future studies to explore this further.

### Strengths and limitations

To the best of our knowledge, the present study is the first population-based study to thoroughly examine the internet addiction (IA) and health literacy (HL) across three dimensions of middle school students. This cross-sectional survey provided sufficient evidence to suggest a negative association between adolescent internet addiction and health literacy levels. According to the present results, interventions aimed at preventing or reducing the rate of IA may be targeted at improving health literacy. It is highly recommended that further research by health education specialists extend on the current findings to explore the moderating and mediating effects of relevant variables on the relationship between internet addiction and health literacy among adolescents. Further studies are necessary to confirm whether adequate health literacy plays a role in the success of internet addiction interventions and the extent to which it is reached.

Nevertheless, this survey provided important evidence regarding health literacy and added evidence to the limited literature on health literacy among middle school students. However, the present study had several limitations. First, the Internet Addiction Diagnostic Scale, which was taken from the Chinese Adolescent Health Risky Behavior Questionnaire (Junior Middle School Part), is widely used among adolescents in China. However, it may not be valid to compare the rates of IA among different countries due to the difficulty of measuring IA. In addition, the present study only had comparative data and no normative data on IA, indicating that interpretations should be made with caution. Second, the Adolescent Health Literacy Scale was a revision of our original work. We have completed item screening and reliability analysis by surveying 612 students in grades 7–12 in Chongqing. Although the reliability and validity of the initial health literacy scale for middle school students were high, no paper has been published on the results of the scale’s assessment, and comparisons with other studies need to be made cautiously due to the different research instruments. Furthermore, the present study had a cross-sectional study design, which made causal relationships undeterminable. Therefore, cohort studies are recommended to gain more insight into this subject.

## Conclusion

The present cross-sectional study investigated the characteristics of internet addicts and the association between HL and IA among middle school students in Chongqing, China. According to the present, the rate of internet addiction (IA) was 6.1%, which was relatively high. IA was strongly associated with critical, functional and interactive health literacy but not total health literacy. Critical health literacy was a protective variable for internet addiction, while functional, interactive health literacy was a risk variable. Moreover, the present findings extended previous research on variables related to internet addiction, and they provided preliminary evidence for the predictive role of health literacy in adolescents’ internet addiction and ideas for health policy-makers and service providers to develop preventive measures related to internet addiction. Further, longitudinal studies are needed to provide more evidence for this hypothesis.

## Supporting information

S1 File The internet addiction diagnostic questionnaire in English. (PDF)

S2 File The adolescent health literacy scale in English. (PDF)

S1 Data (SAV)
