# Supplementary material for: The relationship between health literacy and internet addiction among middle school students in Chongqing, China: A cross-sectional survey study
Source: PLoS One. 2023 Mar 24;18(3):e0283634. doi: 10.1371/journal.pone.0283634 (PMC10038306; doi:10.1371/journal.pone.0283634)
Supplement: S1 File — (PDF) [file pone.0283634.s001.pdf]

Internet Addiction Diagnostic Questionnaire

**1. In the past week, how long did you usually spend online each day (including using mobile phones, iPads and other electronic devices)?**<br>①Less than 1 hour ②1-4 hours (excluding 4 hours) ③4 hours or more ④No Internet access (skip to next table)		
**2. Do you have any of the following situations?**	**Yes**	**No**
(1) Often surfing the internet, the internet-related things keep coming to mind even without going online;		
(2) Feeling uncomfortable or don't want to do other things once you can't surf the internet, which is relieved by going online;		
(3) Increasing the time spent online to get satisfaction;		
(4) Losing interest in other recreational activities (hobbies, meeting friends) for surfing the internet;		
(5) Trying to stop surfing the internet many times, but always cannot control yourself;		
(6) Not being able to finish homework or play truant for surfing the internet;		
(7) Hiding the fact that you surf the internet from parents, teachers or classmates;		
(8) Continuing to surf the internet knowing the negative consequences (lack of sleep, being late for class, arguing with parents);		
(9) Surfing the internet in order to escape from reality, get rid of your dilemmas or depression, helplessness or anxiety.		
**3. Do your parents limit the amount of time you spend watching TV, playing on your mobile phone or playing video games each day?**<br>① Yes ② No		
