# Supplementary material for: The relationship between health literacy and internet addiction among middle school students in Chongqing, China: A cross-sectional survey study
Source: PLoS One. 2023 Mar 24;18(3):e0283634. doi: 10.1371/journal.pone.0283634 (PMC10038306; doi:10.1371/journal.pone.0283634)
Supplement: S2 File — (PDF) [file pone.0283634.s002.pdf]

Adolescent Health Literacy Scale

Please answer each question according to your real situation or thoughts, and put a “ √ ” on the corresponding option.

	Strongly disagree	Disagree	Unsure	Agree	Strongly Agree
1. Health is the intact state of physical, mental and social adaptation	1	2	3	4	5
2. Everyone has the responsibility to maintain their own health and the health of others	1	2	3	4	5
3. Healthy lifestyle mainly includes balanced diet, moderate exercise, smoking cessation and alcohol restriction, and psychological balance	1	2	3	4	5
4. A healthy lifestyle can maintain and promote one's health	1	2	3	4	5
5. Appropriate nutrition refers to a diet with a full range of nutrients and scientific proportions	1	2	3	4	5
6. Take medication as prescribed by your doctor and not without instruction	1	2	3	4	5
7. Outdoor activities help prevent myopia	1	2	3	4	5
8. Consume at least 12 kinds of food on average every day	1	2	3	4	5
9. Eat vegetables at every meal, eat fruit every day	1	2	3	4	5
10. Eat a moderate amount of meat every day	1	2	3	4	5
11. Drink one glass (bag) of milk or two bags of yogurt every day	1	2	3	4	5
12. Drinking plain boiled water is healthier than sugary drinks	1	2	3	4	5
13. Eat a nutritious breakfast can improve cognitive ability and reduce the risk of overweight and obesity	1	2	3	4	5
14. Snacks such as nuts and fruit can be eaten in moderation between meals	1	2	3	4	5
15. Vaccination is the most effective measure to prevent some infectious diseases	1	2	3	4	5
16. Tuberculosis is mainly transmitted through coughing and sneezing by tuberculosis patients	1	2	3	4	5
17. Passive smoking can lead to many diseases	1	2	3	4	5
18. Spoiled food can easily cause food poisoning	1	2	3	4	5
19. Balanced diet combined with moderate exercise can effectively prevent overweight or obesity	1	2	3	4	5
20. Maintaining correct reading and writing posture helps prevent myopia	1	2	3	4	5
21. Puberty is the period of gradual transition from childhood to adulthood	1	2	3	4	5
22. Understanding the characteristics of physical development during puberty contributes to healthy growth	1	2	3	4	5
23. Growth of height and weight accelerates during puberty	1	2	3	4	5
24. Spermatorrhea or Menarche is the normal physiological manifestation of puberty	1	2	3	4	5

25. The timing of pubertal development varies among individuals	1	2	3	4	5
26. A positive mindset and good interpersonal relationships contribute to mental health	1	2	3	4	5
27. Seek help from parents or teachers when you are in a bad mood	1	2	3	4	5
28. Adolescents are prone to psychological and behavioral problems	1	2	3	4	5
29. Psychological and behavioral problems may occur when facing academic pressure	1	2	3	4	5
30. Many people have psychological and behavioral problems that they are not aware of	1	2	3	4	5
31. Most psychological and behavioral problems can be solved	1	2	3	4	5
32. Cut off the power quickly and call for help when getting an electric shock	1	2	3	4	5
33. After being bitten by a pet, rinse with water and inject with rabies vaccine immediately	1	2	3	4	5
34. Wounds caused by iron nails should be cleaned with water and injected with tetanus serum immediately	1	2	3	4	5
35. Obey the traffic rules when crossing the road and do not cross the road	1	2	3	4	5
36. Wear seat belts when riding in a car	1	2	3	4	5
37. Do not swim unaccompanied by an adult	1	2	3	4	5
	Completely inconsistent	Inconsistent	Unclear	Consistent	Completely consistent
38.Actively seek medical care and consciously obtain relevant health information when you are sick	1	2	3	4	5
39. Be able to read and understand instructions of drugs	1	2	3	4	5
40. Call 120 when you need emergency medical assistance	1	2	3	4	5
41. Check the shelf life of food every time you buy food	1	2	3	4	5
42. Be able to express your symptoms clearly when you are sick	1	2	3	4	5
43. Ask others for health information that you are not sure	1	2	3	4	5
44. Understand the health information transmitted by healthcare workers	1	2	3	4	5
45. Seek resources around you to help solve health problems	1	2	3	4	5
46. Eat breakfast every day and make sure it is nutritious	1	2	3	4	5
47. Eat a regular, well-balanced diet every day	1	2	3	4	5
48. Sleep no less than 8 hours every day	1	2	3	4	5
49. Exercise for more than 1 hour every day	1	2	3	4	5
50. Do warm-up exercise before physical exercise, and do finishing exercises after strenuous exercise	1	2	3	4	5
51. Children and adolescents should avoid alcohol	1	2	3	4	5
52. Will think about the reliability of health knowledge taught by school teachers	1	2	3	4	5
53. Will think about the reliability of health knowledge transmitted by relatives and friends	1	2	3	4	5

54. Will think about the reliability of health knowledge disseminated online	1	2	3	4	5
55. Will think about the reliability of health knowledge disseminated by newspapers and magazines	1	2	3	4	5
56. Will consider the correctness of “appropriate nutrition is good for health”	1	2	3	4	5
57. Will consider the correctness of "taking medication without permission after illness"	1	2	3	4	5
58. Will consider the correctness of "obeying traffic rules can effectively prevent accidental injuries"	1	2	3	4	5
59. Will consider the correctness of "sharing toothbrushes with others or tattoos carries a risk of contracting AIDS or hepatitis B"	1	2	3	4	5
60. Will judge the applicability of preventing drowning by avoiding approaching dangerous waters	1	2	3	4	5
61. Will judge the applicability of preventing gas poisoning by paying attention to ventilation when using water heaters	1	2	3	4	5
